# Gene-Regulatory Activity of α-Tocopherol

**DOI:** 10.3390/molecules15031746

**Published:** 2010-03-12

**Authors:** Gerald Rimbach, Jennifer Moehring, Patricia Huebbe, John K. Lodge

**Affiliations:** 1 Institute of Human Nutrition and Food Science, Christian Albrechts University 24118 Kiel, Germany; E-Mails: moehring@foodsci.uni-kiel.de (J.M.); huebbe@foodsci.uni-kiel.de (P.H.); 2 Cranfield Health, Vincent Building, Cranfield University, Cranfield, Bedfordshire MK43 0AL, UK; E-Mail: j.k.lodge@cranfield.ac.uk (J.K.L.)

**Keywords:** vitamin E, α-tocopherol, antioxidant, gene expression

## Abstract

Vitamin E is an essential vitamin and a lipid soluble antioxidant, at least, under *in vitro* conditions. The antioxidant properties of vitamin E are exerted through its phenolic hydroxyl group, which donates hydrogen to peroxyl radicals, resulting in the formation of stable lipid species. Beside an antioxidant role, important cell signalling properties of vitamin E have been described. By using gene chip technology we have identified α-tocopherol sensitive molecular targets *in vivo* including christmas factor (involved in the blood coagulation) and 5α-steroid reductase type 1 (catalyzes the conversion of testosterone to 5α-dihydrotestosterone) being upregulated and γ-glutamyl-cysteinyl synthetase (the rate limiting enzyme in GSH synthesis) being downregulated due to α-tocopherol deficiency. α-Tocopherol regulates signal transduction cascades not only at the mRNA but also at the miRNA level since miRNA 122a (involved in lipid metabolism) and miRNA 125b (involved in inflammation) are downregulated by α-tocopherol. Genetic polymorphisms may determine the biological and gene-regulatory activity of α-tocopherol. In this context we have recently shown that genes encoding for proteins involved in peripheral α-tocopherol transport and degradation are significantly affected by the apoE genotype.

## 1. Introduction, Chemistry and Antioxidant Properties of Vitamin E

**Figure 1:**
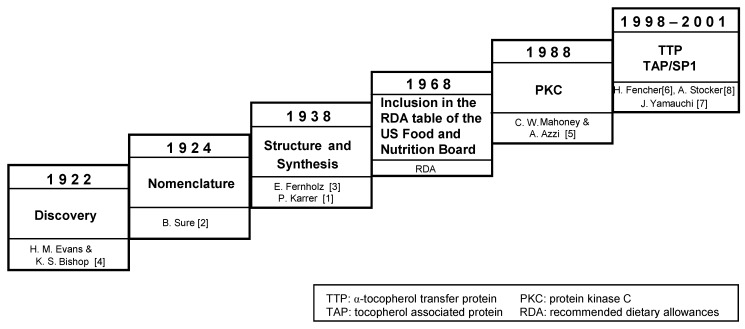
Milestones in vitamin E research.

Vitamin E was discovered at the University of California at Berkeley in 1922 in the laboratory of Herbert M. Evans (Figure 1). Eight vitamin E isoforms with biological activity have been isolated from plant and animal sources. Tocopherols are part of an interlinking set of antioxidant cycles, which has been termed the so-called antioxidant network [9]. Since its discovery, mainly antioxidant and recently also cell signalling aspects of tocopherols have been studied. Advances in gene chip and miRNA technology have led to the discovery of novel vitamin E-sensitive genes and vitamin E regulated signal transduction pathways. Polymorphisms in genes involved in tocopherol tissue uptake, export, and metabolism may be important determinants for the biological activity of vitamin E. 

**Figure 2:**
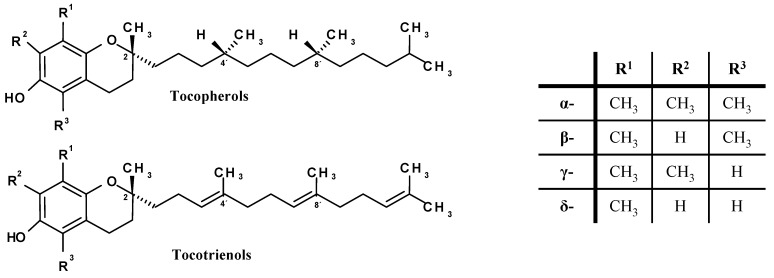
Molecular structure of vitamin E stereoisomers.

Vitamin E consists of a mixture of tocopherols and tocotrienols that are synthesised by plants from homogenestic acid. All are derivatives of 6-chromanol with an aliphatic side-chain. The four tocopherol homologues (α-, β-, γ-, δ-) have a fully saturated 16-carbon isoprenoid side-chain, whereas tocotrienols have a similar isoprenoid chain containing three double bonds. Individual tocopherols are named according to the position and number of the methyl groups on the phenol ring, with the α-, β-, γ- and δ- vitamins containing three, two, two and one methyl groups respectively (Figure 2). These structural differences determine the biological activity, with α-homologues being the most biologically active [10]. 

The antioxidant property of vitamin E is exerted through the phenolic hydroxyl group, which readily donates its hydrogen to the peroxyl radical, resulting in the formation of a stable lipid species. In donating the hydrogen atom, vitamin E becomes a relatively unreactive free radical as the unpaired electron becomes delocalised into the aromatic ring. The efficiency of this protection depends upon two factors: firstly the mobility of the molecule in membranes, which is determined by the aliphatic tail; secondly the number of methyl species on the chromanol ring, with each methyl group conferring additional antioxidant capacity. In addition, the proximity of the methyl species to the hydroxyl group is an important factor. Therefore α-homologues, which have the greatest number of methyl species, and in which these flank the hydroxyl group, are thought to be more effective than the other homologues. Nevertheless, in several *in vitro* studies a reverse order of antioxidant efficacy with α-tocopherol being the least potent compound as compared to δ- and γ-tocopherol has been observed [11,12,13,14,15]. Furthermore, in superiority to α-tocopherol, γ-tocopherol and other non-α-tocopherols are able to trap membrane-soluble electrophilic nitrogen oxides and other electrophilic mutagens possessing free aromatic ring positions and thereby more efficiently inhibiting reactive nitrogen species derived damage [16,17].

**Figure 3:**
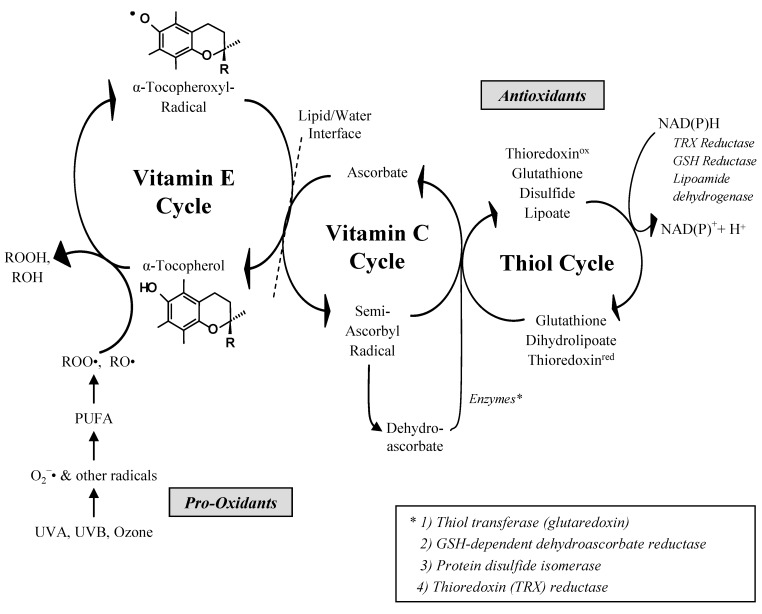
The antioxidant network showing the interaction among vitamin E, vitamin C and thiol redox cycles (according to [9]) © American Society for Nutrition); PUFA: polyunsaturated fatty acid, ROOH: lipid peroxide, ROH: alcohol, ROO•: peroxyl radical; RO•: alkoxyl radical, O_2_•^−^: superoxide anion radical, NAD(P)H: nicotinamide-adenine-dinucleotide-(phosphate).

Vitamin E does not work in isolation from other antioxidants; rather it is part of an interlinking set of redox antioxidants, as summarized in Figure 3. The antioxidant activity of vitamin E has been detailed many times (e.g., [18]). In this regard vitamin E is able to scavenge peroxyl radicals essentially neutralizing them to form hydroperoxides. Lipid peroxyl radicals are formed during lipid peroxidation. Following an initiating event induced by, for example, a reactive oxygen species, a hydrogen molecule is abstracted from a C-H bond that has been weakened due to the proximity to an electron withdrawing double bond found in polyunsaturated fatty acids forming a carbon-centered radical. During this process there is molecular rearrangement of the lipid to a conjugated diene structure and addition of O_2_ to the carbon-centered radical giving rise to a lipid peroxyl radical. This radical is highly reactive and if not quenched will react with a nearby polyunsaturated fatty acid causing another initiating event and hence propagating lipid peroxidation. As a scavenger of peroxyl radicals vitamin E is acting to inhibit this chain reaction which is why it is termed a chain-breaking lipid antioxidant. Without vitamin E activity a single initiating event could potentially lead to the production of thousands of lipid peroxides which would have a deleterious effect on the biological membrane and its function. The sequence of events can be seen as:

(1) -LH + ROS → L
(2) (-L^.^ + O_2_ → -LOO^.^
(3) -LOO^.^ + VEH → LOOH + VE^.^

It is hypothesized that vitamin E acts catalytically, being efficiently reduced from its free radical (chromanoxyl) form that arises after quenching lipid radicals to return back to its reduced native state. This catalysis occurs through the interactions between water- and lipid-soluble substances by both nonenzymatic and enzymatic mechanisms that regenerate vitamin E from its tocotrienoxyl or tocopheroxyl radical back to tocotrienol and tocopherol respectively. Vitamin C can regenerate vitamin E directly, and thiol antioxidants, such as glutathione and lipoic acid, can regenerate vitamin E indirectly via vitamin C. Under conditions where these systems act synergistically to keep the steady-state concentration of vitamin E radicals low, the loss or consumption of vitamin E is prevented [9].

Previous studies in cultured cells indicate synergistic effects between polyphenols and vitamin E (γ-tocopherol but not α-tocopherol) *in vitro* [19]. However, dietary supplementation of growing pigs with green tea polyphenols did not affect serum, liver, lung and muscle vitamin E (α- and γ-tocopherol) concentrations, plasma antioxidant capacity [20]. Furthermore, also in rats [21] and humans [22] no vitamin E (α- and γ-tocopherol) sparing effect of dietary flavonoids was evident.

## 2. Gene Regulatory Activity of Vitamin E

**Figure 4:**
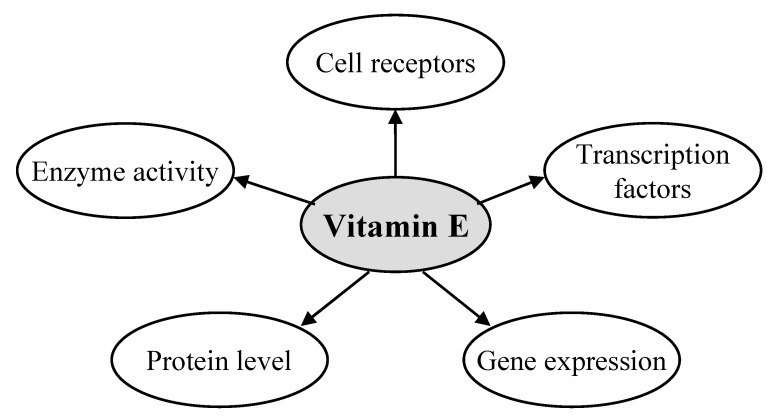
Vitamin E is a cell signalling molecule and affects cell receptors, transcription factors, gene expression, protein levels and enzyme activities of vitamin E specific molecular targets.

Since its discovery, mainly antioxidant and recently also cell signalling aspects of tocopherols have been studied. Vitamin E is a cell signalling molecule [23]. It interacts with cell receptors (e.g., LDL-receptor) and transcription factors (e.g., pregnane X receptor) thereby driving (redox-regulated) gene expression (e.g., scavenger receptor CD36). It modulates protein levels (e.g., glutathione) and changes enzyme activity levels (e.g., protein kinase C) both in cultured cells as well as *in vivo* (Figure 4). 

Azzi's group [5,24,25] first described non-antioxidant cell signalling functions for α-tocopherol, demonstrating that vitamin E inhibits protein kinase C (PKC) and 5-lipoxygenase and activates protein phosphatase 2A and diacylglycerol kinase. Some genes including α-tocopherol transfer protein (α-TTP), α-tropomyosin, and collagenase are affected by α-tocopherol at the transcriptional level.

To obtain a comprehensive understanding of the molecular mechanisms of action of vitamin E, global gene expression profile experiments using DNA arrays in rat liver and hepatocellular liver carcinoma cells (HepG2) have been conducted. 

For the analysis of short-term (49 days) [26] and long-term (290 days) vitamin E deficiency [27], rats were fed semisynthetic diets either supplemented with or deficient in vitamin E (DL-α-tocopheryl acetate [26]; *RRR*-α-tocopheryl acetate [27]). In addition, HepG2 cells were treated with vitamin E (*RRR*-α-tocopheryl acetate) concentrations comparable to those that were achieved in the *in vivo* experiment [28]. Differential gene expression in rat liver and that in HepG2 cells were measured by DNA arrays comprising up to 7,000 genes. Dietary vitamin E deficiency over a 7-week period did not induce any significant changes in the expression profile among the genes evaluated. However, long-term vitamin E deficiency upregulated coagulation factor IX (FIX), 5-α-steroid reductase type 1, and CD36 mRNA levels. Furthermore, vitamin E deficiency resulted in a significant downregulation of hepatic γ-glutamyl-cysteinyl synthetase, the rate-limiting enzyme of glutathione synthesis. Measurement of the corresponding biological endpoints such as activated partial thromboplastin time, plasma dihydrotestosterone and hepatic glutathione substantiated the gene chip data which indicated that dietary vitamin E plays an important role in a range of metabolic processes within the liver. According to the rat experiment, vitamin E supplementation changed coagulation factor IX and CD36 expression in HepG2 cells; thus, *in vivo* data could be partly confirmed with the *in vitro* model. Overall, our studies reveal that dietary vitamin E has important long-term effects on liver gene expression with potential downstream effects on extrahepatic tissues.

We have recently studied the short- and long-term effects of natural and synthetic vitamin E on cytochrome (CYP) P450 dependent gene expression using Affymetrix GeneChip technology. To this end, HepG2 hepatoma cells were incubated with 0, 10, 30, 80 and 300 μM *RRR*-tocopheryl acetate (natural vitamin E) or *all-rac*-tocopheryl acetate (synthetic vitamin E) for 7 days and the mRNA of CYP genes was quantified. The expression of only one (CYP20A1) of 14 CYP genes with detectable mRNA levels was dosedependently up-regulated. No differences in gene-regulatory activity were observed between *RRR*- and *all-rac*-α-tocopheryl acetate. To study the role of vitamin E in CYP gene expression *in vivo*, Fisher 344 rats were randomly assigned to either a vitamin E-enriched (*RRR*-tocopheryl acetate) or –deficient diet. Neither in the vitamin E-enriched, nor in the vitamin E-deficient rats, were significant changes in the liver CYP mRNA levels observed. These data indicate that vitamin E did not appear to modulate cytochrome P450 mRNA expression in our HepG2 cells or our in rat study [29]. However, other studies demonstrate that α-tocopherol supplementation did increase mRNA level and activity of several CYP members in the liver of mice and rats [30,31].

In another set of experiment two groups of male rats were fed with either a diet deficient in α-tocopherol or a control diet enriched with *RRR*-α-tocopheryl acetate. Differential gene expression in skeletal muscle was monitored at five time-points over 430 days, with all animals individually profiled [32]. Out of approximately 7,000 genes represented on the gene chip, 56 were found to be up-regulated in response to vitamin E in at least four consecutive time-points from as early as 91 days of deficiency. Up-regulated genes included muscle structure and extra cellular matrix genes, as well as anti-oxidative, anti-inflammatory and anti-fibrotic genes. Our data show that molecular transcription might provide a very early marker to detect oncoming degenerative conditions in vitamin E deficiency. They provide further insight into possible molecular mechanisms underlying vitamin E deficiency in skeletal muscle, and reveal the activation of an intensive protection program that can explain the long maintenance of muscle structure during vitamin E deficiency.

Furthermore in rat brain a significant number of genes were found to be regulated by vitamin E. These vitamin E sensitive genes encode for proteins associated with hormones and hormone metabolism, nerve growth, apoptosis, dopaminergic neurotransmission, and clearance of amyloid-β and advanced glycated endproducts [33].

**Figure 5:**
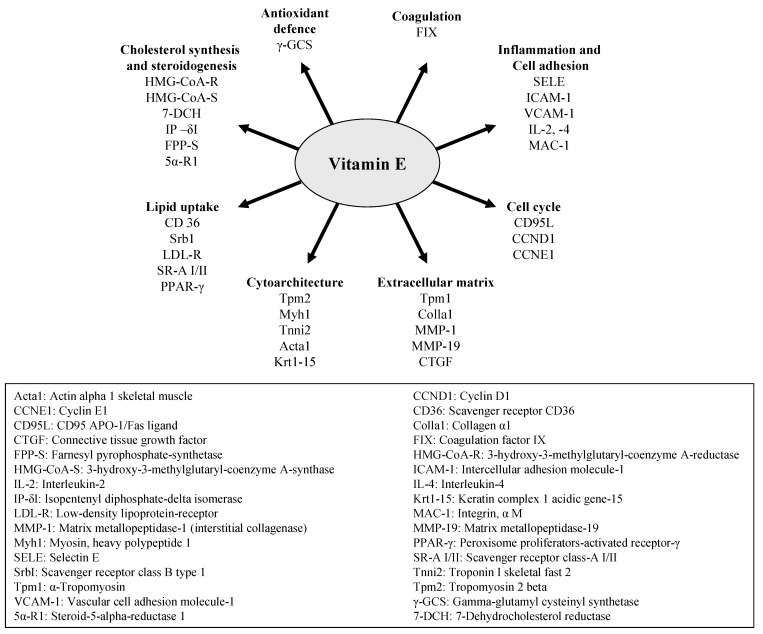
Transcriptional regulation of vitamin E target genes.

Our *in vivo* data in rats also indicate that dietary vitamin E (*RRR*-α-tocopheryl acetate) induces changes in steroidogenesis by affecting cholesterol homeostasis in testes and adrenal glands. In this context genes encoding for proteins involved in the uptake (LDL receptor) and de novo synthesis (e.g., 7-dehydrocholesterol reductase, 3-hydroxy-3-methylgluteryl coenzyme A synthase, 3-hydroxy-3-methylglutaryl coenzyme A reductase, isopentenyl diphosphate δ-isomerase, and farnesyl pyrophosphate synthetase) of cholesterol (Figure 5), the precursor of all steroid hormones, have been identified as vitamin E sensitive molecular targets [34].

## 3. Comparative Quantification of Pharmacodynamic Parameters of *RRR*- *vs*. *all-rac*-α Tocopherol by Global Gene Expression Profiling

Pharmacologically active compounds (e.g., from the groups of pharmaceutical drugs, cofactors or vitamins) often consist of two or more stereoisomers (enantiomers or diastereoisomers) which may differ in their pharmacodynamic/kinetic, toxicological and biological properties. A well-known example is vitamin E which is predominantly administered as two different forms, one derived from natural sources (mainly soybeans), and one from production by chemical total-synthesis. While vitamin E from natural sources occurs as a single stereoisomer (*RRR*-α-tocopherol), synthetic vitamin E (*all-rac*-α-tocopherol) is an equimolar mixture of eight stereoisomers. Based on a number of animal and human studies it has been suggested that the biological potency of natural-source vitamin E (*RRR-*) is 1.36 greater compared to its counterpart produced by chemical synthesis (*all-rac*) [35]. We have used the Affymetrix GeneChip technology to evaluate the feasibility of a new bio-assay where the gene regulatory activities of *RRR*-α-tocopherol and *all-rac*-α-tocopherol were quantified and compared on the genome-wide level [36]. For this purpose, HepG2 cells were supplemented with increasing amounts of *RRR*- or *all-rac*-α-tocopherol for seven days. Genes showing a dose-related induction/repression were identified by global gene expression profiling. Our findings show that *RRR*- and *all-rac*-α-tocopherol share an identical transcriptional activity, *i.e.,* induce/repress the expression of the same set of genes. Based on the transcriptional dose-response data, EC50 and IC50 values were determined for each of these genes. The feasibility of calculating a "transcriptional potency factor" of *RRR*- vs. *all-rac*-α-tocopherol was evaluated by dividing the EC50/IC50 of *RRR*-α-tocopherol by the corresponding EC50/IC50 of *all-rac*-α-tocopherol for every of the vitamin E responsive genes. Using this approach we have calculated 215 single biopotency ratios. Subsequently, the mean of all potency ratios was found to be 1.05. 

Biopotency in humans has been the subject of some debate. Several biokinetic studies comparing the relative bioavailability of natural and synthetic vitamin E in humans used a competitive uptake approach whereby both deuterated *RRR*- and *all-rac*-α-tocopheryl acetate were administered simultaneously [37,38,39]. Using this competitive approach bioavailability ratios of *RRR:all-rac* close to 2:1 were found. Using a non-competitive approach, whereby deuterated *RRR-* and *all-rac*-α-tocopheryl acetate are administered on separate occasions to the same individual, bioavailability ratios, C_max_ and AUC analysis, (the important parameters of bioavailability in single dose studies), were 1.35:1, 1.35:1 and 1.33:1 respectively [40], very close to the accepted biopotency ratio of 1.36:1 derived from animal studies [41,42] and in stark contrast to the findings of studies using the competitive uptake approach. It has been argued that the competitive uptake model results in bias against *all-rac*, because when *RRR* and *all-rac* are given simultaneously in equal amounts, the actual dose would contain 75 % of 2*R*-forms to compete with 25 % of 2*S*-forms. As the 2*R*-forms are preferentially selected by α-TTP for systemic distribution, increasing the abundance of 2*R*-forms lowers the probability of α-TTP binding to 2*S*-forms leading to discrimination against *all-rac* [43]. However when *all-rac* is administered separately to *RRR*, there would be 50 % each of the 2*R*- to 2*S*-forms in *all-rac* and hence no such discrimination would occur. Therefore when studied using a non-competitive approach under basal conditions, natural (*RRR*) and synthetic (*all-rac*) α-tocopherol are kinetically equivalent and their relative bioavailability conforms to the currently accepted biopotency ratio of 1.36:1 [40].

## 4. Vitamin E Regulated miRNAs

miRNA are a class of small, noncoding RNA, that in their mature form, are single-stranded and 22 nucleotides long. Mature miRNA post-transcriptionally affect gene expression by binding at the 3´ untranslated region of mRNA and inhibiting their translation into proteins [44]. It has been suggested that each miRNA binds on average 100 different target mRNA, allowing for post-transcriptional silencing of many different genes, or potentially entire pathways, by a single miRNA [45]. miRNA are encoded for in the genome and are, thus, liable to regulation. In order to investigate the role of dietary *RRR*-α-tocopherol on miRNA expression, we fed rats for 6 months with diets deficient or sufficient in vitamin E and analyzed miRNA concentrations in the liver. For this study, we selected miRNA that were previously shown to be involved in processes that have been associated with vitamin E, namely lipid metabolism (miRNA-122a) [46,47], cancer progression and inflammation (miRNA-125b) [48,49,50]. Vitamin E deficiency resulted in decreased levels of miRNA-122a and miRNA-125b. Apart from its role in lipid metabolism, miRNA-122 was down-regulated in rodent and human hepatocellular carcinomas [47]. A role for miRNA-125b in cancer development has been suggested because miRNA-125b was down-regulated in human prostate cancer tissues [48,49], lung cancer cell lines [51], breast cancer [52], and in squamous cell carcinoma of the tongue [53]. Shi and colleagues [54], on the other hand, found over-expression of miRNA-125b in prostate cancer cell lines and prostate cancer samples.

A role of miRNA-125b in inflammation is supported by a study that identified tumor necrosis factor-alpha (TNFα) as a direct target of miRNA-125b. A decrease of miRNA-125b resulted in increased TNFα production and inflammation in LPS stimulated macrophages [55]. Thus, the reduced miRNA-125b levels observed in our vitamin E-deficient rats may be associated with an enhanced inflammatory response due to vitamin E deficiency, as previously described [56,57]. Overall these data indicate that vitamin E regulates cell signalling not only at the mRNA level but also at the miRNA level [58]. Although gene chip and micro-RNA technology has helped to identify vitamin E sensitive molecular targets, transcription factors which are specifically regulated by vitamin E have yet not been identified. Furthermore a receptor that specifically binds to vitamin E is currently unknown [59].

## 5. Single Nucleotide Polymorphisms in Genes Important in Vitamin E Homeostasis

Large inter-individual variation exists in the response to vitamin E supplementation, and this may influence the outcome of human studies. It is our hypothesis that genetic heterogeneity is an important determinant of vitamin E homeostasis. Therefore we have performed an *in silico* search for single nucleotide polymorphisms (SNPs) associated with genes involved in vitamin E homeostasis. Based on function, the following genes were considered as candidates for vitamin E heterogeneity: α-tocopherol transfer protein (α-TTP), tocopherol associated protein (TAP), lipoprotein lipase (LPL), multidrug resistance protein 2 (MDR-2), pregnane X receptor (PXR) and members of the cytochrome P450 family (CYP). Searches for coding SNPs were initiated from web based programs of the National Center for Biotechnology Information (NCBI). SNP frequencies were calculated by dividing the number of annotated coding SNPs by the number of base pairs in the open reading frame. Genes for α-TTP, TAP and CYP3A5 had calculated SNP frequencies between 503 and 837 base pairs per coding SNP (bp/cSNP) and so are not highly polymorphic. In contrast, cSNP frequencies in LPL, MRP2, PXR, CYP3A4 and CYP4F2 were in the range of 100 bp/cSNP and so are highly polymorphic. Thus proteins involved in specific vitamin E binding are not highly polymorphic, may not influence inter-individual variation and so may not be good candidates for population studies. Proteins involved in drug/lipid metabolism which indirectly influence vitamin E status are highly polymorphic, are likely to influence inter-individual variation and so are good candidates for population studies. In this context it has been shown in a biokinetic study that subjects with the apoE4 genotype differ from apoE3 subjects in their plasma uptake of newly absorbed α-tocopherol [60] indicating that this is an important SNP influencing vitamin E status. We suggest that future studies are aimed at addressing the role of such SNPs in vitamin E homeostasis [61].

## 6. ApoE—Vitamin E Interactions

In Westernised societies the apoE4 genotype is associated with increased morbidity and mortality, and represents a significant risk factor for cardiovascular disease, late-onset Alzheimer's disease and other chronic disorders. ApoE is an important modulator of many stages of lipoprotein metabolism and traditionally the increased risk was attributed to higher lipid levels in E4 carriers. However, more recent evidence demonstrates the multifunctional nature of the apoE protein and the fact that the impact of genotype on disease risk may be in large part due to an impact on oxidative status or the immunomodulatory/anti-inflammatory properties of apoE. An increasing number of studies in cell lines [62,63], transgenic rodents [64] and human volunteers [65,66] indicate higher oxidative stress and a more pro-inflammatory state associated with the epsilon4 allele [67]. Natural antioxidants including dietary vitamin E may counteract the adverse affect of the apoE4 genotype on oxidative stress and chronic inflammation.

**Figure 6:**
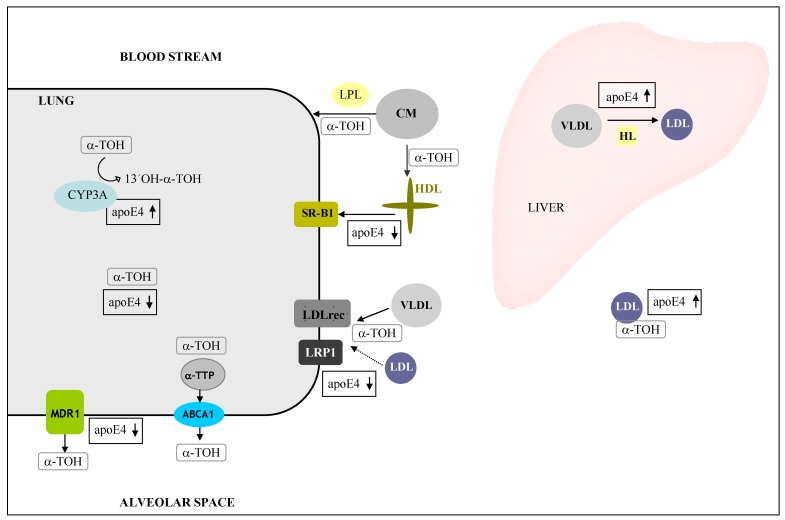
Proteins involved in the metabolism of α-tocopherol (α-TOH) in the lung. α -tocopherol is distributed in the body via different lipoproteins and chylomicrons (CM). Cellular uptake of α-TOH is mediated by apoE and lipoprotein lipase (LPL) activity and their binding to several lipoprotein receptors including peripheral tissues (e.g., lung)receptor (LDLrec) and LDL receptor related protein 1(LRP1). Intracellular α-TOH is subjected to mitochondrial degradation by the cytochrome P450 family 3A (CYP3A) enzyme subfamily. Delivery of α-TOH to the alveolar space is facilitated by the activity of the α-tocopherol transfer protein (α-TTP) and ATP-binding cassette A1 (ABCA1). Excessive amounts of α-TOH may be also excluded via the multidrug resistance transporter 1 (MDR1) (according to [68]).

In a recent study, we observed significant lower α-tocopherol concentrations in the lung of apoE4 compared with apoE3 transgenic mice. Genes encoding for proteins involved in peripheral α-tocopherol transport degradation (Figure 6) are affected by the apoE genotype probably accounting for the lower α-tocopherol tissue concentration as observed in apoE4 mice. Infact, receptors of α-tocopherol uptake including scavenger receptor B1 (SR-B1), LDL receptor (LDLrec) and LDL receptor related protein 1 (LRP1) were lower in apoE4 as compared to apoE3 mice. Lung mRNA levels of the ATP-binding cassette A1 (ABCA1) and the multidrug resistance transporter 1 (MDR1), surfactant proteins mediating the export of α-tocopherol, were lower in apoE4 than in apoE3 mice. In addition, the mRNA levels of cytochrome P450 3A (CYP3A), an enzyme family involved in the degradation of α-tocopherol, tended to be higher in the apoE4 as compared to the apoE3 genotype. Taken together our data indicate that the apoE4 may retain less α-tocopherol in the lung than apoE3 genotype, which may in turn impact on the dietary vitamin E requirement. 

Similar to the apoE4 polymorphism, the haptoglobin 2-2 polymorphism is associated with increased production of reactive oxygen species and decreased tissue levels of vitamin E and importantly, vitamin E supplementation (d-α-tocopherol) appears to reduce cardiovascular events in individuals with diabetes mellitus type 2 and the Hp 2-2 genotype [69].

In this context a so-called personalized nutritional advice may be taken into account where individuals could be given dietary recommendations in terms of vitamin E considering their apoE and haptoglobin genotype [70].

The use of ‘-omic’technologies, some of which has already been discussed in this article, has advanced the notion of personalized nutrition. Metabolomics, a study of the complement of metabolites in biological samples, has been viewed as having an important role in the development of a nutritional phenotype for future personalized nutritional assessment [71,72]. Metabolomics offers the ability to determine how nutrients interact with metabolic pathways [71,73] offering insights into the complex relationships between diet and metabolism and diet and disease and therefore maybe able to define novel mechanisms of action. Both targeted and nontargeted metabolite profiling approaches can be used to study vitamin E status [74]. Targeted approaches to understand vitamin E physiology and metabolism include the use of stable isotope labeled vitamin E which allows researchers to identify factors that influence vitamin E status in humans including dietary, genetic, lifestyle and metabolic factors [35,75]. Nontargeted metabolomics is used to investigate global metabolite profiles in order to identify novel targets. Interestingly this approach has previously been used to investigate vitamin E status in murine models. Griffin *et al*. studied the metabolic deficits and vitamin E deficiency induced by neuronal ceroid lipofuscinosis in a mouse model using metabolomics and revealed how vitamin E supplementation could partially reverse some of the metabolic abnormalities [76]. More recently Cho *et al*. demonstrated that activation of the pregnane X receptor attenuated vitamin E metabolism to the usual carboxyethyl hydroxychroman (CEHC) form but discovered a novel vitamin E metabolite [77]. We have previously highlighted that metabolomics can be used to provide insights into how vitamin E can influence the human metabolome [74,75] and indeed demonstrated some interesting observations following a supplementation regime that are undergoing confirmation. In this regard it may be possible to identify novel mechanisms of vitamin E action and greatly expand our knowledge of the activities of vitamin E.
